# Development of a multi-year white-nose syndrome mitigation strategy using antifungal volatile organic compounds

**DOI:** 10.1371/journal.pone.0278603

**Published:** 2022-12-01

**Authors:** Kyle T. Gabriel, Ashley G. McDonald, Kelly E. Lutsch, Peter E. Pattavina, Katrina M. Morris, Emily A. Ferrall, Sidney A. Crow, Christopher T. Cornelison

**Affiliations:** 1 Department of Molecular and Cellular Biology, Kennesaw State University, Kennesaw, Georgia, United States of America; 2 United States Fish and Wildlife Service, Ecological Services, Athens, Georgia, United States of America; 3 Georgia Department of Natural Resources, Wildlife Resources Division, Wildlife Conservation Section, Social Circle, Georgia, United States of America; 4 Department of Biology, Georgia State University, Atlanta, Georgia, United States of America; University of Jeddah, SAUDI ARABIA

## Abstract

*Pseudogymnoascus destructans* is a fungal pathogen responsible for a deadly disease among North American bats known as white-nose syndrome (WNS). Since detection of WNS in the United States in 2006, its rapid spread and high mortality has challenged development of treatment and prevention methods, a significant objective for wildlife management agencies. In an effort to mitigate precipitous declines in bat populations due to WNS, we have developed and implemented a multi-year mitigation strategy at Black Diamond Tunnel (BDT), Georgia, singly known as one of the most substantial winter colony sites for tricolored bats (*Perimyotis subflavus*), with pre-WNS abundance exceeding 5000 individuals. Our mitigation approach involved *in situ* treatment of bats at the colony level through aerosol distribution of antifungal volatile organic compounds (VOCs) that demonstrated an *in vitro* ability to inhibit *P*. *destructans* conidia germination and mycelial growth through contact-independent exposure. The VOCs evaluated have been identified from microbes inhabiting naturally-occurring fungistatic soils and endophytic fungi. These VOCs are of low toxicity to mammals and have been observed to elicit antagonism of *P*. *destructans* at low gaseous concentrations. Cumulatively, our observations resolved no detrimental impact on bat behavior or health, yet indicated a potential for attenuation of WNS related declines at BDT and demonstrated the feasibility of this novel disease management approach.

## Introduction

Emerging fungal diseases (EFDs) have become a growing concern worldwide for biodiversity and food security, with several plant and animal pathogens posing direct threats to ecosystems and food production [1]. One particular fungal pathogen has recently been introduced to naive species of bats in North America, and if left unchecked, threatens certain bat species with population decline, extirpation, and extinction [2]. *Pseudogymnoascus destructans* [3–5] is a psychrophilic ascomycete that has been identified as the etiological agent responsible for the deadly EFD among North American bats known as white-nose syndrome (WNS) [5,6]. WNS is typified by an invasion of bat tissue by *P*. *destructans* during torpor, a state of severely reduced metabolic activity that enables survival through the winter season. During torpor, the bat’s metabolic activity, including temperature and immune function, is greatly reduced. While torpid, the body temperatures of bats often fall within the growth range of *P*. *destructans* (0°C to 20°C) [7]. Coupled with suppressed immune function, clinical WNS can quickly develop. The resulting tissue damage from fungal invasion is known to cause the disruption of thermoregulation, water regulation, and electrolyte balance, as well as increase the rate of torpor arousal that leads to a return to euthermia, resulting in premature expenditure of fat stores crucial to survive winter, among other effects [8–10]. Additionally, immune reconstitution inflammatory syndrome may exacerbate tissue damage following arousal from torpor [11]. These insults have led to severe population declines for several species, with up to 99% mortality in just a few years following the pathogen’s introduction to some hibernacula [2], as well as shifts in bat assemblage [12].

The rapid spread and high mortality associated with WNS has made development of methods for treating and preventing *P*. *destructans* infections an important objective for wildlife management agencies. Accordingly, the development of biological and chemical treatment methods has become a significant priority for State and Federal agencies, as outlined in the 2011 National WNS Management Plan established by the United States Fish and Wildlife Service [13].

A number of potential mitigation methods have been developed or experimentally tested against *P*. *destructans* or treating white-nose syndrome, including chemical agents [14–20], microbial antagonists [21–27], environmental modulation [28,29], UV light exposure [30–32], antibiotics [33], vaccination [34], and electrolyte supplementation [35], among others. Of these, the greatest interest has been in the use of chemical and microbial agents to inhibit the growth and pathogenicity of *P*. *destructans*.

The fungistatic properties of suppressive soils have been widely observed across terrestrial environments [36], with volatile organic compounds (VOCs) being largely responsible for these antimicrobial activities. These chemicals typically have low molecular weights, high vapor pressures, and readily evaporate at standard temperature and pressure, and are of particular interest due to their ability to inhibit microbial growth in dense and diverse ecosystems from a gaseous, contact-independent exposure [37–42]. Recent research has investigated microbially-produced VOCs for their potential agricultural benefits, by preventing infection through controlling pathogens and stimulating crop growth [43,44].

Our early investigations demonstrated the ability of a ubiquitous soil-dwelling bacterium, *Rhodococcus Rhodochrous* strain DAP 96253, to inhibit the growth of several fungal pathogens and spoilage organisms, including *P*. *destructans*, when placed in a shared airspace with the target organism [22,45]. Further investigations utilizing antimicrobial VOCs demonstrated the feasibility of their use *in vitro* to suppress *P*. *destructans* conidia germination and mycelial growth [14]. The low inhibitory concentrations observed with select VOCs suggested a potential to utilize these compounds in novel ways to combat undesired microbial growth and infection. Furthermore, this methodology precludes the handling of bats and enables treatment at the colony level, reducing both labor and disturbance. Accordingly, we developed a multi-year mitigation strategy at Black Diamond Tunnel to test the feasibility of a VOC-mediated, *in situ* treatment of bats.

**Fig 1:**
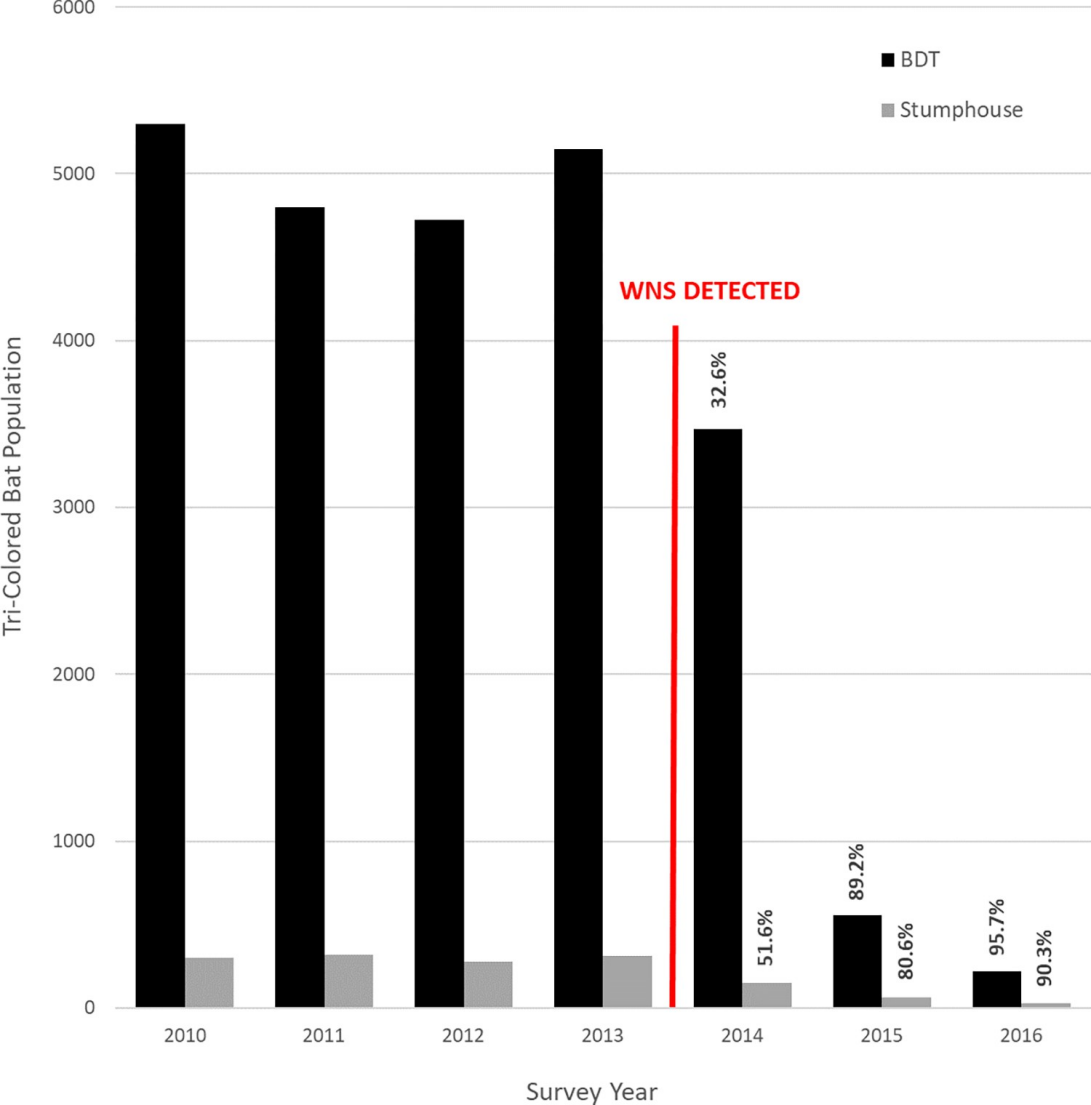
Bat populations at Black Diamond Tunnel and Stumphouse Tunnel from 2010 to 2016. Yearly tricolored census at Black Diamond Tunnel in Clayton, Georgia, and Stumphouse Tunnel in Oconee County, South Carolina. The red line indicates when WNS was first detected. Percentages indicate the percent decline at each tunnel since WNS was detected.

Black Diamond Tunnel is an abandoned railway tunnel in Clayton, Georgia, in the United States, and was singly known as one of the most substantial winter colony sites for tricolored bats (*Perimyotis subflavus*), with abundance exceeding 5,000 individuals in the years prior to 2014 (Fig 1). Construction of the tunnel began in the 1850s but was halted in the 1860s due to the American Civil War, and was subsequently never resumed, leaving the tunnel uncompleted and abandoned. Black Diamond Tunnel has a single entrance approximately 4.4 meters in width and 5.4 meters in height, and extends 423 meters straight into the rock mountainside at a slight downward slope. As a result of slow drainage, water has filled the entire length of the tunnel. The tunnel’s environmental stability, protection from predators, positioning on the landscape where very few caves are present, and large fresh water source were likely driving factors that established it as a significant bat hibernaculum. Following the detection of WNS in 2013, the tricolored bat colony at BDT experienced a precipitous decline, with greater than 95% mortality observed over the following 3 years. A comparative site to BDT is Stumphouse Tunnel, Oconee County, SC, situated ten air miles from BDT. The conditions of the tunnels differ, with Stumphouse Tunnel being of smaller volume and having less water and fewer bats, however it is the closest hibernaculum to BDT that is regularly surveyed and will serve as an untreated hibernaculum for comparison to BDT (Fig 1).

Many of the chemicals and formulations chosen for evaluation were identified from previously-published literature that analyzed the gaseous headspace constituents of suppressive soils and cultures of microorganisms that have demonstrated microbial antagonism. *Muscodor crispans* strain B-23 is one such microorganism, a novel endophytic fungus isolated from a Bolivian wild pineapple (*Ananas ananassoides*), that has demonstrated an ability to inhibit several plant and human pathogens via contact-independent antagonism [46,47]. A synthetic formulation of the gaseous compounds produced by *Muscodor crispans* strain B-23 [48] has been reproduced under the commercial name Flavorzon 185B (Jeneil Biotech, Inc., Saukville, WI, USA) and only includes compounds listed by the food and drug administration as generally regarded as safe (GRAS). This synthetic formulation, further referred to as B-23, has been demonstrated as safe and of low toxicity to mammals, and has been incorporated into several commercial products for use with multiple mammal species, agriculture, and food storage [49]. Several qualities of B-23 make it appealing to use as a treatment agent, including its ability to inhibit the growth of *P*. *destructans* (Quist, unpublished bachelor’s thesis), observations of bats demonstrating a non-aversion to B-23-soaked sachets (Last and Morris, unpublished 2014 WNS Workshop presentation), and its constituents listed as GRAS.

Beginning in the winter of 2016, BDT became the site of our ongoing evaluation of a VOC-based WNS mitigation effort using the L-30 electric rotary atomizer (Curtis Dyna-Fog, Ltd., Westfield, IN, USA) to distribute antifungal VOC formulations. The goals of this effort were to demonstrate the feasibility of a VOC-based mitigation system to treat bats at a colony level and to improve the health and survivorship of bats affected by WNS. To this end, we have demonstrated the feasibility of this novel disease management approach as well as a potential attenuation of WNS related declines at BDT.

## Materials and methods

### Treatment chemical formulation development

Several antimicrobial susceptibility assays with synthetically-produced chemicals and formulations were conducted previously in order to discover novel antagonistic and synergistic effects against *P*. *destructans* using aliquots of VOCs dispensed on absorbent discs and sealed in a shared airspace with *P*. *destructans*-inoculated media [14].

Presently, *P*. *destructans* antagonism was further evaluated based on a 24-hour gaseous exposure to antifungal formulations to simulate an environment where 100% of aerosolized treatment formulation had evaporated to a gas. *P*. *destructans* was grown for 2 weeks at 15°C from a 100 μl conidia solution (10^6^ conidia ml^-1^) spread on a 90 mm Petri dish of Sabouraud dextrose agar (SDA, BD Difco, Becton, Dickinson and Company, New Jersey, USA) to produce a confluent lawn. Six millimeter diameter mycelial plugs were removed from the confluent lawn of *P*. *destructans* with transfer tubes (Spectrum Laboratories, Massachusetts, USA) and inserted into the center of sterile SDA in 30 mm Petri dishes. A liquid amount of the B-23 formulation was completely evaporated using a hot plate in order to attain a specific gaseous concentration within a sealed glove box. The inoculated 30 mm Petri dishes were placed open inside larger 150 mm Petri dishes, then double sealed with paraffin film to capture the gaseous concentration of the glove box within the headspace of the large Petri dish. A series of gaseous concentrations (100, 300, and 500 ppmv) were captured in separate Petri dishes. All trials were conducted in triplicate and incubated a 15°C for 24 hours before being opened to evacuate the headspace of antifungal chemicals. All Petri dishes were then resealed and incubated at 15°C for 2 weeks. Area growth was measured per the methods of Cornelison et al. [14] and experimental groups were compared to controls to determine the degree of inhibition elicited from the exposure.

### Treatment dispersal device development

Several aerosolization technologies were evaluated for compatibility with the treatment methodology. Dispersal rate, acoustic output, aerosol droplet diameter, and portability were the primary considerations when selecting the dispersal device for our application. Dispersal rates were determined by measuring the amount of water dispersed from the device after a defined period of operation. Acoustic output was measured with an ultrasonic microphone (M500, Pettersson Elektronik, Uppsala, Sweden) from a distance of 10 feet in front of the device where chemicals were discharged. Aerosol droplet diameters were referenced from the specifications listed in the literature provided by the manufacturers. Portability was qualitatively graded based on weight, size, and design, which included the presence of handles, durability, power source, and mounting options. Dispersal devices evaluated included a jet nebulizer (Trek S, PARI Respiratory Equipment Inc., Germany), blower aerosolizers (Cyclone Ultra II and Hurricane Ultra II, Curtis Dyna-Fog Ltd., Westfield, IN, USA), and the L-30 rotary atomizer (Curtis Dyna-Fog Ltd., Westfield, IN).

### Infrastructure development

**Fig 2:**
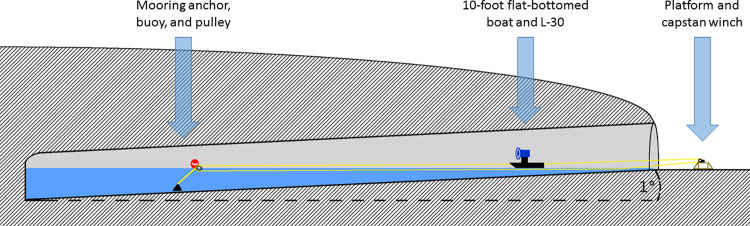
Diagram of the infrastructure at Black Diamond Tunnel.

To prepare the BDT site for treatment, infrastructure was erected inside and outside of the tunnel to enable movement of the dispersal device across the tunnel’s water surface (Fig 2). A platform was constructed to secure a battery-powered capstan winch (Powerwinch 300; Powerwinch, Colorado, USA) that enabled extension and retrieval of a 10-foot flat-bottomed boat containing the L-30 rotary atomizer. A pulley was attached to a buoy and secured near the rear of the tunnel with 20 feet of 0.25-inch diameter polypropylene rope to a mooring anchor. A 600-meter length of 0.25-inch diameter braided polypropylene rope was fed though the pulley, the free ends were attached to carabiners with eye splices, then attached to the bow and stern of the boat. With the open loop wrapped around the capstan winch drum, the vessel was able to be moved in and out of the tunnel at will by actuating the capstan winch at the entrance. Demarcations on the rope every 12 meters permitted accurate determination of the position of the boat inside the tunnel.

### Black Diamond Tunnel treatment calculations

Accurately calculation of the amount of treatment formulation to attain a specific gaseous concentration in BDT required estimating the airspace volume of the tunnel. Two sets of height and width measurements of the tunnel were acquired using a laser measurement device (GLM 30 laser measure, Bosch, Germany) while traversing the tunnel on boat, with effort made to acquire measurements at a consistent interval. Each set of height and width measurements was used to create a volumetric profile of the tunnel. Three different volumetric calculation methods were employed. The first method summed the volumes calculated from adjacent area measurements. The second and third method applied either a linear and third-degree polynomial trend line to the height and width data prior to calculating and summing the volumes, in an attempt to reduce miscalculations resulting from measurement inaccuracies.

Calculations were used to determining the amount of the treatment formulation needed to attain specific gaseous concentrations. The molar gaseous volume of a substance is first calculated with the ideal gas law:

$$V_{I}=\frac{RT}{P}$$ (1)

Where *V*_*I*_ is the volume of the gas (L), *R* is the Gas Constant 8.314462 J K^−1^ mol^−1^, *T* is the temperature (Kelvin), and *P* is the pressure (kPa). The total amount *A* of the compound (mg) is then calculated:

$$A=\frac{M}{V_{I}}∙V_{G}∙C$$ (2)

Where *M* is the molecular weight of the compound (g/mol), *V*_*G*_ is the volume of the airspace (m^3^), and *C* is the desired gaseous concentration (ppmv). This was repeated for each compound of the formulation. Attaining a 300 ppmv gaseous concentration of B-23 in a 5,300 m^3^ airspace at 5°C and 100 kPa, assuming complete volatilization, was calculated to require 6.65 liters of liquid B-23 formulation. The addition of decanal to the B-23 formulation, at a 1:1 ratio and increasing the treatment concentration to 500 ppmv, yielded 16.32 liters total, comprised of 5.542 liters B-23 and 10.782 liters decanal. The L-30 was calibrated to disperse at 3.3 x 10^−3^ L s^-1^, rendering a total run time of 2,015 seconds to disperse 6.65 liters and 4,945 seconds to disperse 16.32 liters.

The vessel with the L-30 atomizer was winched 300 meters into the tunnel prior to beginning dispersal. This was the furthest point at which safe operation could be achieved without risking contacting the L-30 with the ceiling of the tunnel. To promote a homogeneous dispersal throughout the tunnel, the vessel retrieval duration was synchronized with the dispersal duration. Two retrieval methods were evaluated to accomplish this synchronization.

The first method employed an electric winch operating at a 100% duty cycle, resulting in a vessels speed of 0.43 m s^-1^, and a movement of 300 meters in 698 seconds. With the L-30 requiring a longer duration to disperse the target treatment volume, the vessel was delayed the additional time by periodically pausing its movement every 12-meter demarcation on the rope for an appropriate amount of time.

The second method employed a DC motor speed controller to modulate the duty cycle of the winch motor in order to slow the vessel speed to match the retrieval duration with the dispersal duration. Winch speed was empirically determined to have a linear relationship with duty cycle, enabling the determination of the appropriate duty cycle to attain a desired speed:

$$W=\frac{1000∙S+343}{4571}$$ (3)

Where *W* is the winch duty cycle (%) and *S* is the desired speed (m s^-1^). Additionally, the load of the boat and atomizer was found to have a negligible effect on winch speed.

The entrance of the tunnel was not sealed for any of the treatments in order to allow bats to freely leave the tunnel and to permit a visual assessment of bat disturbance. Treatments were conducted in early November, December and January of each winter season from 2016 to 2022, during the period when bats are known to experience infection and disease symptoms and hypothesized to be the period when VOC treatment can potentially have the greatest impact on reducing *P*. *destructans* growth and bat mortality. Bat population surveys were conducted prior to each first and second treatment of the season, as well as a pre-arousal survey in late February.

### Environmental sampling

On December 2, 2016 we collected pre-treatment and one-hour post-treatment water samples at the tunnel entrance. We collected air samples 150 meters from the tunnel entrance, immediately following dispersal of 300 ppmv B-23. We collected analogous air and water samples 7 days post-treatment at the same locations. Samples were analyzed using gas chromatography-mass spectrometry to determine the presence of the treatment formulation at each time point.

### Bat colony census

In comparison to natural caves and extensive mines, where many wintering bats choose to roost, BDT lacks complexity, existing as a linear excavation through metamorphic schist. These characteristics allow for bats to be easily observed and identified. The tunnel can be surveyed throughout its entire 423 meter length, unlike other winter roosts that may have bats roosting in inaccessible passages and crevices. Prior to VOC treatments, annual censuses of BDT were conducted by the US Fish and Wildlife Service (USFWS) and Georgia Department of Natural Resources (GSDNR), consisting of two counting observers and a rowboat operator. Each observer employed portable spotlights and hand-held tally counters, with each observer counting their designated side of the tunnel to avoid double-counting bats. A complete census required approximately one-hour.

When we initiated VOC treatments at BDT, we monitored the response of tricolored bats to the potential disturbance from the rotary atomizer, as well as the antifungal compounds dispersed during fumigation, with the decision to immediately suspend VOC treatments if we observed behavioral responses from the bat colony, such as abandonment of the roost, arousal during treatment, or bats flying at the tunnel entrance. To employ this strategy, USFWS and GADNR performed three winter census efforts: (1) early winter census (late October—early November), prior to the VOC treatment; (2) mid-winter census (early-mid December), subsequent to the first, seasonal VOC treatment but prior to the second, seasonal VOC treatment; and (3) a late winter census (late February—early March) that corresponded to statewide WNS census and surveillance efforts and matched prior censuses at BDT dating back to 2010. Qualitative observations were recorded of the bat colony as well as notations on clinical presentation of WNS such as visible fungus on wings and muzzle, consistent with field signs, as detailed in WNS case definitions set forth by the WNS National Plan Diagnostic Working Group [50]. Observations were limited to visual inspection only in order to limit handling and disturbance to the tricolored bat colony, and to reduce the risk of individual bats drowning if they were removed from their roosting locations. Additionally, methods of analyzing WNS infection with the use of UV transillumination as a non-destructive diagnostic technique [51] was not employed.

Black Diamond Tunnel is privately owned and access was granted by the property owner via a license to Georgia State University, which was transferred to Kennesaw State University in 2017. Bat handling was approved by the KSU Institutional Animal Care and Use Committee under protocol #21–002. A 2021 biological evaluation was completed as part of the National Environmental Policy Act compliance, where normal surveys were deemed not likely to adversely affect listed species. Since the USFWS was a cooperator and all treatments at Black Diamond were under USFWS supervision at all times, work was performed under the USFWS Federal Permit (NATIVE ENDANGERED SP. RECOVERY—ENDANGERED WILDLIFE; ENDANGERED PLANTS; MIGRATORY BIRDS). Additionally, in the biological evaluation for the project, it was determined that effects to threatened northern long-eared myotis was unlikely because the species has likely extirpated from the site, being in excess of five years since it’s been observed.

## Results and discussion

### Treatment chemical formulation development

After a 24 hours of gaseous exposure of B-23 to *P*. *destructans*, inhibition of mycelial growth was observed with a concentration as low as 300 ppmv (unpublished data). Decanal, a GRAS compound previously-determined to be effective at inhibiting *P*. *destructans*, was later included in treatment formulation development because of its low mammalian toxicity and its ability to inhibit mycelial growth and conidia germination (Cornelison et al., 2014a).

An initial target treatment concentration of 300 ppmv B-23 was chosen for BDT, as this was the minimal amount determined *in vitro* to elicit *P*. *destructans* inhibition. After the first season of treatments, both the VOC formulation constituents and the dispersal concentration was modified in an effort to improve treatment efficacy, with the new formulation consisting of B-23 and decanal (Sigma Aldrich, St. Louis, MO, USA), at a 1:1 ratio and the treatment concentration increased to 500 ppmv.

### Treatment dispersal device development

Several aerosol-producing devices were evaluated for their suitability for our application, including a jet nebulizer, blower aerosolizers, and the L-30 rotary atomizer. Droplet size was a significant consideration in device selection due to the physical properties of spheres and evaporation. As an aerosol droplet diameter decreases, its surface-area-to-volume ratio increases, thereby increasing the total surface area of the aerosol, facilitating evaporation to the gaseous phase. Smaller aerosol droplets also have increased hang-time, reducing deposition on surfaces and prolonging exposure to the air to further promote evaporation.

**Fig 3:**
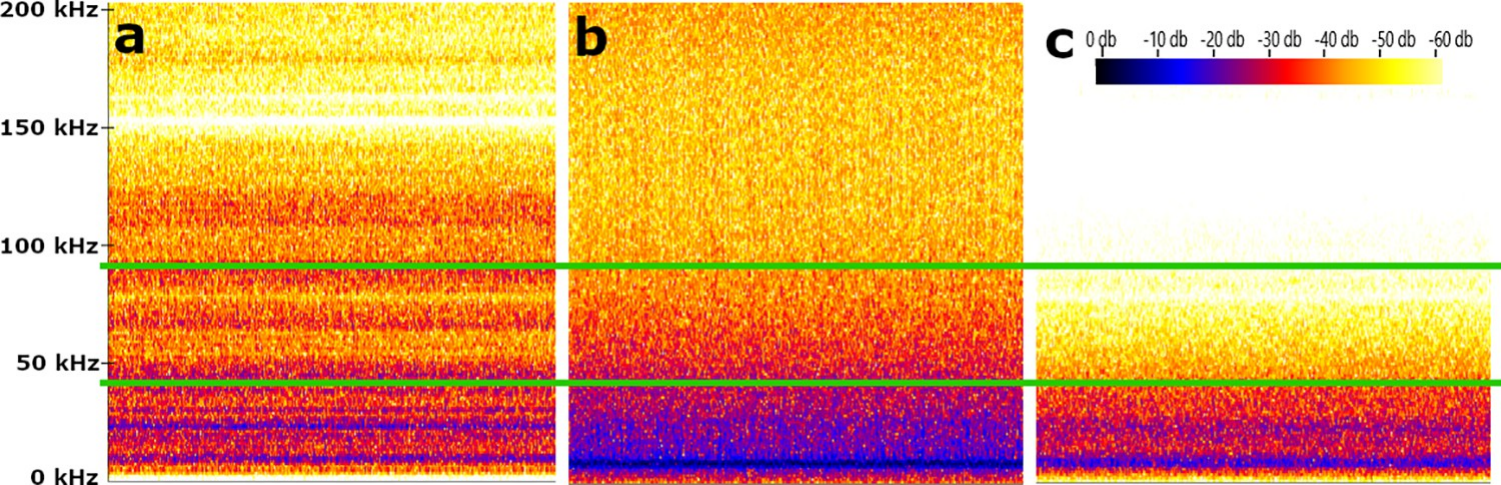
Aerosolizer acoustic output. Acoustic output of a jet nebulizer (a), blower aerosolizer (b), and L-30 rotary atomizer (c). The x-axis is time and the y-axis is frequency in kHz. Amplitude is represented on a spectrum with louder measurements being darker. The green bars identify the common range *P*. *subflavus* uses for echolocation (45–90 kHz).

Although the jet nebulizer tested produced the smallest droplet size (3–5 volume mean diameter, VMD), the dispersal rate (0.1–2 ml min^-1^) was insufficient to attain the desired concentration in BDT in a reasonable amount of time. Additionally, the jet nebulizer tested emitted high frequencies in the range that could potentially disturb bats and interfere with echolocation (0–200 kHz). The blower aerosolizers tested produced an adequate droplet size (5–20 VMD) but subpar dispersal rate (0–70 ml min^-1^) and also produced high frequency output (0–200 kHz). Of all devices tested, the L-30 rotary atomizer produced an adequate droplet size (24 VMD), dispersal rate (10–200 ml min^-1^), and emitted the lowest frequency output (0–80 kHz) and the lowest amplitude in the *P*. *subflavus* echolocation range (Fig 3).

### Airspace volume of Black Diamond Tunnel

Due to the variability of boat movement speed, distance between measurements were unable to be precisely determined, however volume calculations were conducted with the assumption that measurements were at evenly-spaced intervals. Three methods were used to calculate the air volume in the tunnel: segments from the raw data, segments from linear trends of the raw data, and segments from 3° polynomial trends of the raw data. An estimated tunnel volume of approximately 5,300 m^3^ was established.

### Environmental sampling

Gas chromatography-mass spectrometry (GCMS) analysis of air samples obtained 1-hour post-treatment yielded several chromatographic peaks that were in agreement with a 300 ppmv gas standard, however, abundance varied. This indicates environmental factors at BDT might affect air movement dynamics, volatility, and gaseous homogeneity, among other factors. GCMS analysis of the 7-day post-treatment air samples indicated there was significantly less B-23 present than 1 hour post-treatment samples. GCMS analysis of the 1-hour and 7-day post-treatment water samples could not detect any constituents of the B-23 formulation.

### Black Diamond Tunnel treatment and bat colony census

Bat colony censuses were conducted for 6 years prior to beginning treatments at BDT (Table 1). Treatment dates, chemical formulations, concentrations, and tricolored bats counted are listed on Table 2.

**Table 1:** Pre-treatment *Perimyotis subflavus* censuses and observations.

Date	Live	Dead	Notable Observations
2010 Jan. 25	5300	NA	Pre-WNS invasion.
2011 Mar. 2	4800	NA	Pre-WNS invasion. Diagnostic swabs collected with negative confirmation of fungal pathogen.
2012 Jan. 26	4723	0	Pre-WNS invasion. Diagnostic swabs collected with negative confirmation of fungal pathogen.
2013 Feb. 26	5148	0	Pre-WNS invasion. Diagnostic swabs collected with negative confirmation of fungal pathogen.
2014 Mar. 4	3472	>88	Large WNS mortality event with approx. 15% tricolored bats exhibiting field signs of WNS. *P*. *destructans* presence confirmed via swabs. Many bats observed dead within hibernaculum, especially near the entrance.
2015 Mar. 12	554		Post-WNS invasion with 75% of bats exhibiting field signs of WNS.
2016 Mar. 1	220		

**Table 2:** Dates of treatments and *Perimyotis subflavus* censuses with information about specific formulation and concentration.

Date	VOC Formulation	VOC Concentration	Live Tricolored Bats
2016 Oct. 20			204
2016 Nov. 4	B-23	300 ppmv	206
2016 Dec. 2	B-23	300 ppmv	216
2017 Jan. 6	B-23	300 ppmv	
2017 Feb. 28			152
2017 Oct. 20			204
2017 Nov. 3	B-23	500 ppmv	
2017 Dec. 4	B-23	300 ppmv	271
2018 Jan. 3	B-23	300 ppmv	
2018 Feb. 28			178
2018 Nov. 9	B-23 + decanal	500 ppmv	190
2018 Dec. 4	B-23 + decanal	500 ppmv	183
2019 Jan. 25	B-23 + decanal	500 ppmv	
2019 Feb. 28			179
2019 Nov. 15	B-23 + decanal	500 ppmv	219
2019 Dec. 12			217
2019 Dec. 13	B-23 + decanal	500 ppmv	
2020 Jan. 14	B-23 + decanal	500 ppmv	
2020 Feb. 27			216
2020 Nov. 13			227
2020 Nov. 18	B-23 + decanal	500 ppmv	
2020 Dec. 18	B-23 + decanal	500 ppmv	
2021 Jan. 11			255
2021 Jan. 13	B-23 + decanal	500 ppmv	
2021 Feb. 21			272
2021 Nov. 3			387
2021 Nov. 5	B-23 + decanal	500 ppmv	
2021 Dec. 3			338
2021 Dec. 10	B-23 + decanal	500 ppmv	
2022 Jan. 25			362
2022 Jan. 26	B-23 + decanal	500 ppmv	
2022 Feb. 22			364

An initial bat population survey on November 4, 2016, prior to the first treatment, yielded 206 tricolored bats. During the November 4 treatment, no bats were observed arousing or leaving the tunnel. Prior to the second treatment on December 2, 2016, a bat population census was conducted that yielded a count of 216 tricolored bats. Additionally, all bats appeared healthy, with no visual symptoms of WNS present at that time. It was also observed that many of the bats were roosting toward the rear of the tunnel. This second treatment was also conducted without observing any bat arousal or aversion, either during or after treatment. A bat population census was not obtained prior to the January 6, 2017 treatment. A bat population census was conducted February 28, 2017, which yielded a count of 152 bats. It was also noted that the majority of bats were roosting further back into the tunnel and did not appear to present severe symptoms of infection.

On two occasions, across all treatment applications, a single bat was observed arousing and flying in the tunnel and out onto the landscape before returning to roost in BDT. This coincided with treatment application and is attributed to acoustic disturbance. Cumulatively, the infrequent observation of bats arousing during treatment applications is assumed to indicate that the setup and breakdown of the dispersal system, dispersal of the treatment formulation, and the use of the capstan winch were not significantly disruptive to this hibernating population of *P*. *subflavus*.

**Fig 4:**
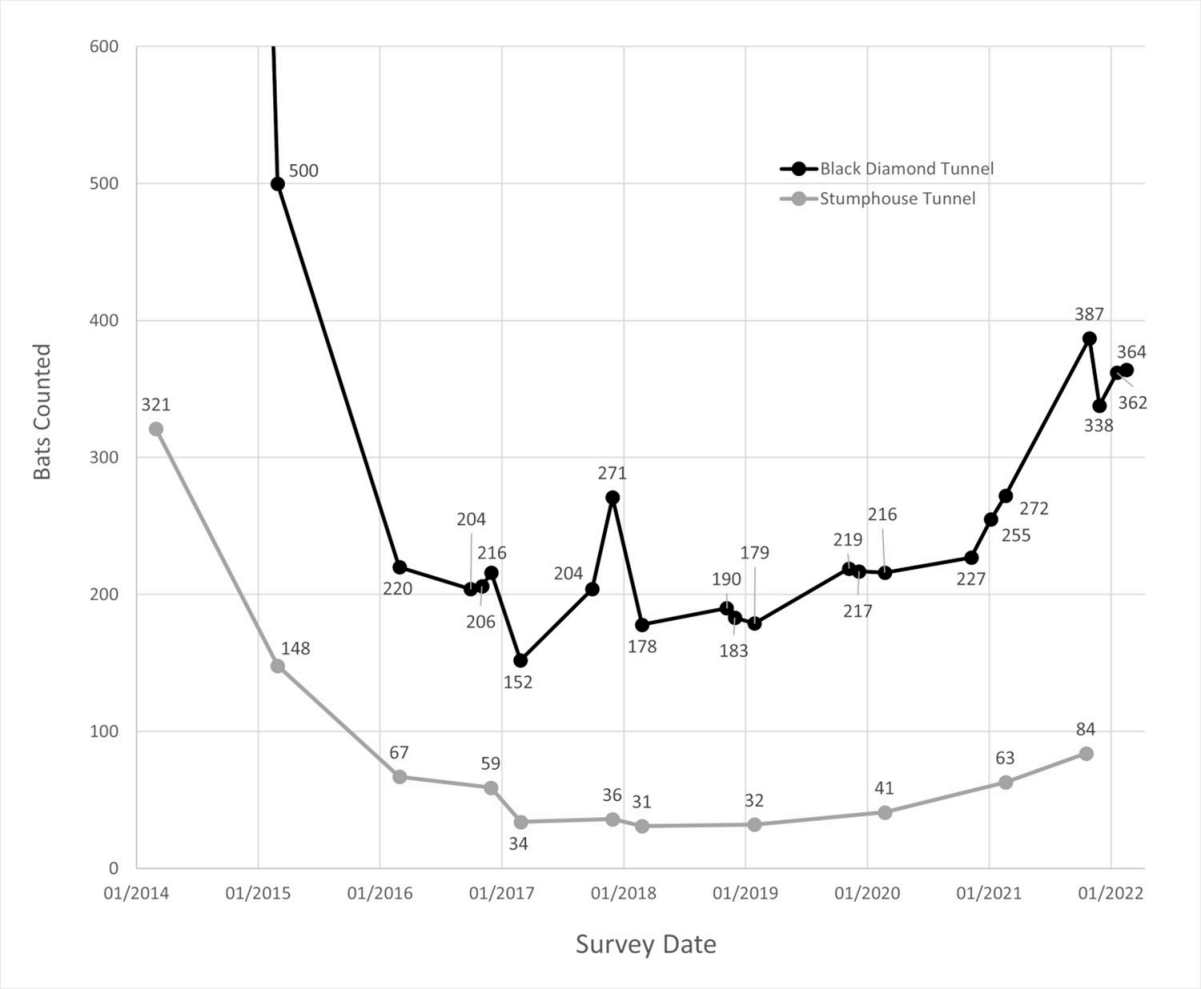
Comparison of bat population censuses at Black Diamond Tunnel and Stumphouse Tunnel.

The population censuses from 2014 to 2021 at Black Diamond Tunnel and Stumphouse Tunnel can be found in Fig 4 (unpublished Stumphouse census data provided by Susan Loeb).

## Conclusion

The contact-independent activity of antagonistic VOCs poses several advantages over contact-dependent treatment options that have been shown to be effective at inhibiting the growth of *P*. *destructans* in previous studies. Treating a hibernaculum using the methods described enables rapid colony-level treatment, reducing both labor and disturbance. Although there is not enough evidence to make any strong conclusions as to whether the treatments at Black Diamond Tunnel were effective at increasing bat survivorship, there were several observations that appear promising. Additionally, determining the cause(s) for the overall observed population stabilization and increase are out of the scope of this project, but these could include reduction of disease severity, reproduction, and migration, among others. The application of these methods in other hibernacula that have greater complexity, such as natural cave environments, will introduce unique challenges that may hinder application and make distribution of treatment formulation through these structures more difficult. Cumulatively, our observations resolved no detrimental impact on bat behavior or health, yet indicated a potential for attenuation of WNS related declines at BDT and demonstrated the feasibility of this novel disease management approach.
